# Paxillin promotes colorectal tumor invasion and poor patient outcomes via ERK-mediated stabilization of Bcl-2 protein by phosphorylation at Serine 87

**DOI:** 10.18632/oncotarget.3537

**Published:** 2015-03-12

**Authors:** Chi-Chou Huang, De-Wei Wu, Po-Lin Lin, Huei Lee

**Affiliations:** ^1^ School of Medicine, Chung Shan Medical University, Taichung, Taiwan; ^2^ Department of Surgery, Division of Colon and Rectum, Chung Shan Medical University Hospital, Taichung, Taiwan; ^3^ Graduate Institute of Cancer Biology and Drug Discovery, Taipei Medical University, Taipei, Taiwan; ^4^ Institute of Medicine, Chung Shan Medical University, Taichung, Taiwan

**Keywords:** paxillin, Bcl-2 phosphorylation, prognosis, colorectal cancer

## Abstract

Stabilization of Bcl-2 protein by paxillin (PXN)-mediated ERK activation was recently reported to cause an unfavorable response to 5-Fluorouracil-based chemotherapy. Here, we present evidence from cell and animal models to demonstrate that stabilization of Bcl-2 protein by phosphorylation at Serine 87 (pBcl-2-S87) via PXN-mediated ERK activation is responsible for cancer cell invasiveness and occurs via upregulation of MMP2 expression. Immunostainings of 190 tumors resected from colorectal cancer patients indicated that PXN expression was positively correlated with Bcl-2, pBcl-2-S87, and MMP2 expression. A positive correlation of pBcl-2-S87 with Bcl-2 and MMP2 was also observed in this study population. Patients with high PXN, Bcl-2, pBcl-2-S87, and MMP2 had poor overall survival (OS) and shorter relapse free survival (RFS). In conclusion, PXN promotes Bcl-2 phosphorylation at Serine 87 via PXN-mediated ERK activation, and its stabilization associated with increased tumor formation efficacy in mice and poor patient outcome in colorectal cancer patients.

## INTRODUCTION

A large body of evidence from tumor specimens now indicates that Bcl-2 expression may be related to favorable prognosis in colorectal cancer [1-4]. Support for this idea comes from a study indicating that the presence of the phosphorylated form of Bcl-2 (pBcl-2-S70, phosphorylated at Serine 70) was linked to tumor regression and patients with favorable outcomes [5]. This was because pBcl-2-S70 accelerated apoptosis via inhibition of the interaction between Bcl-2 and Bax [6]. Stabilization of Bcl-2 protein by phosphorylation at Serine 87 (pBcl-2-S87) plays a predominate role in the increase in Bcl-2 expression [7]. An increase in Bcl-2 expression may promote tumor invasion via increased MMP2 expression [8]. The role of Bcl-2 in tumor progression and patient outcome therefore remains controversial.

Paxillin (PXN) functions as an adaptor protein that recruits diverse cytoskeleton and signaling proteins into a complex and coordinates the transmission of downstream signaling, such as phosphorylation of PXN at Y31 and Y118, which can activate Src and ERK signaling pathways [9, 10]. A recent study has shown that PXN promotes cell proliferation and inhibits apoptosis in SW480 colon cancer cells [11]. Moreover, PXN promotes tumor progression and poor outcome in colorectal cancer patients via decreased miR-137 expression [12]. We reported that stabilization of Bcl-2 protein via phosphorylation of Bcl-2 at Serine 87 (pBcl-2-S87) by PXN-mediated ERK activation conferred 5-fluorouracil (5-FU) resistance and an unfavorable response to 5-FU-based chemotherapy in colorectal cancer patients [13]. In addition, Bcl-2 protein stability by PXN overexpression may partially contribute to PXN-mediated invasion and consequently to cause poorer survival in colorectal cancer patients who received 5-FU-based chemotherapy [13]. However, the underlying mechanism of PXN effects on tumor invasion and poor outcome in colorectal cancer patients is not fully understood.

In the present study, we used cell and animal models to test the possibility that stabilization of Bcl-2 protein by phosphorylation at Serine 87 by PXN-mediated ERK activation may be responsible for tumor progression and metastasis via upregulation of MMP2 expression. We also used Kaplan-Meier and Cox regression models to examine whether pBcl-2-S87 expression in tumors from 190 colorectal cancer patients could be associated with PXN, Bcl-2, and MMP2 expression and whether pBcl-2-S87 expression had a prognostic significance regarding overall survival (OS) and relapse free survival (RFS) in these patients.

## RESULTS

### Increased Bcl-2 protein stability due to ERK-mediated Bcl-2 phosphorylation at Serine 87 is responsible for PXN-mediated cell invasiveness

**Figure 1:**
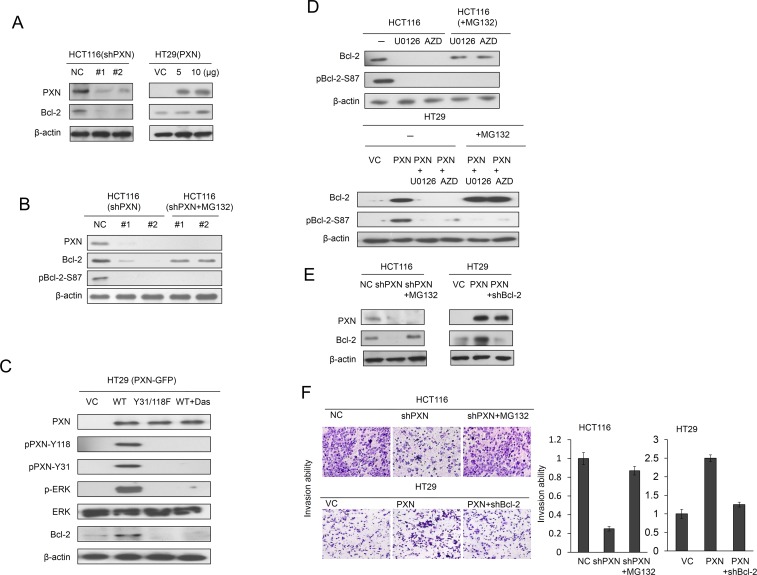
Increased stabilization of Bcl-2 protein by PXN overexpression promotes cell invasion in colorectal cancer cells (A) HCT116 cells were transfected with two different PXN shRNAs. HT29 cells were transfected with two doses of WT PXN and cell lysates were separated by SDS-PAGE to evaluate Bcl-2 and PXN expression by western blotting using specific antibodies. (B) HCT116 cells were transfected with two different PXN shRNAs and then treated with 0 or 5 μmol/L MG132 for an additional 5 hours. Cell lysates were separated by SDS-PAGE to evaluate Bcl-2, pBcl-2-S87, and PXN expression by western blotting using specific antibodies. VC: vector control; NC: non-specific shRNA control. (C) HT29 cells were transfected with WT PXN or mutant PXN-Y31/118F for 36 hours and then treated with 0 or 0.2 μmol/L Src inhibitor (Dasatinib) for an additional 5 hours. The cells lysates were separated by SDS-PAGE to evaluate PXN, pPXN-Y31, pPXN-Y118, ERK, p-ERK, and Bcl-2 expression by western blotting using specific antibodies. (D) HCT116 and PXN-overexpressing HT29 cells were treated with two ERK inhibitors (10 μmol/L U0126 or 5 μmol/L AZD6244, AZD) for 5 hours. These cells were treated with 0 or 5 μmol/L MG132, and then cells were lysed. The cell lysates were separated by SDS-PAGE to evaluate Bcl-2 and pBcl-2-S87 expression by western blotting using specific antibodies. (E) HCT116 cells were transfected with PXN shRNA and then treated with 0 or 5 μmol/L MG132 for an additional 5 hours. HT29 cells were co-transfected with WT PXN and shBcl-2. Cell lysates were separated by SDS-PAGE to evaluate Bcl-2 and PXN expression by western blotting using specific antibodies. (F) Representative numbers of invading HCT116 and HT29 cells transfected with the indicated combinations of WT PXN, shPXN, or shBcl-2. The invasion capability is summarized for the indicated cells.

We explored the possibility that PXN promoted cell invasiveness via increased Bcl-2 protein stability via ERK-mediated Bcl-2 phosphorylation at Serine 87. High and low PXN-expressing HCT116 and HT29 cells were enrolled for knockdown and overexpression of PXN using its small hairpin (sh)RNAs and its expression vector. Bcl-2 expression was markedly decreased by PXN-knockdown in HCT116 cells when compared with HCT116 cells with non-specific shRNA transfection (NC) (Figure 1A left panel). Conversely, Bcl-2 expression was increased in a dose-dependent manner by PXN-overexpression in HT29 cells (Figure 1A right panel). Interestingly, Bcl-2 and pBcl-2-S87 expression were diminished by PXN-knockdown in HCT116 cells (Figure 1B left panel), but the decrease in Bcl-2 expression by PXN-knockdown was rescued by MG132 treatment (Figure 1B right panel). However, no restoration of pBcl-2-S87 was seen in PXN-knockdown HCT116 cells treated with MG132 (Figure 1B right panel).

We further demonstrated that the increase in Bcl-2 by PXN overexpression in HT29 cells was predominately through Src/ERK activation via phosphorylation of PXN at Y31 and Y118. This was due to PXN-mediated Bcl-2 expression, which was not observed in HT29 cells transfected with mutant Y31/Y118F PXN or treated with a Src inhibitor (Figure 1C). The expression of Bcl-2 and pBcl-2-S87 was nearly abolished by a Src or ERK inhibitor; however, Bcl-2 expression was only rescued by MG132 treatment in HCT116 cells (Figure 1D upper panel). Similar results were observed for PXN-overexpressing HT29 cells subjected to the same treatments (Figure 1D lower panel).

We next examined the possibility that PXN-mediated stabilization of Bcl-2 protein due to ERK-mediated Bcl-2 phosphorylation at Serine 87 could be responsible for cell invasiveness. Western blotting indicated that Bcl-2 expression was reduced by PXN-knockdown and elevated by PXN-overexpression, but Bcl-2 expression was rescued by MG132 or Bcl-2 silencing in PXN-knockdown HCT116 and PXN-overexpressing HT29 VC cells (Figure 1E). Boyden chamber assays indicated that The invasion capability was markedly reduced by PXN-knockdown in HCT116 cells and elevated by PXN-overexpression in HT29 cells. The decrease in cell invasiveness by PXN-knockdown was almost completely rescued by MG132 treatment of HCT116 cells (Figure 1F). Conversely, an increase in cell invasiveness by PXN-overexpression was almost completely restored in HT29 cells with Bcl-2 silencing (Figure 1F). Representative invasive cells recovered on Matrigel membranes are shown in Figure 1F (left panel). These results clearly indicate that stabilization of the Bcl-2 protein by phosphorylation at Serine 87 via PXN-mediated ERK activation is responsible for cell invasiveness in colon cancer cells.

### An increase in MMP2 expression by Bcl-2 is responsible for PXN-mediated cell invasiveness

**Figure 2:**
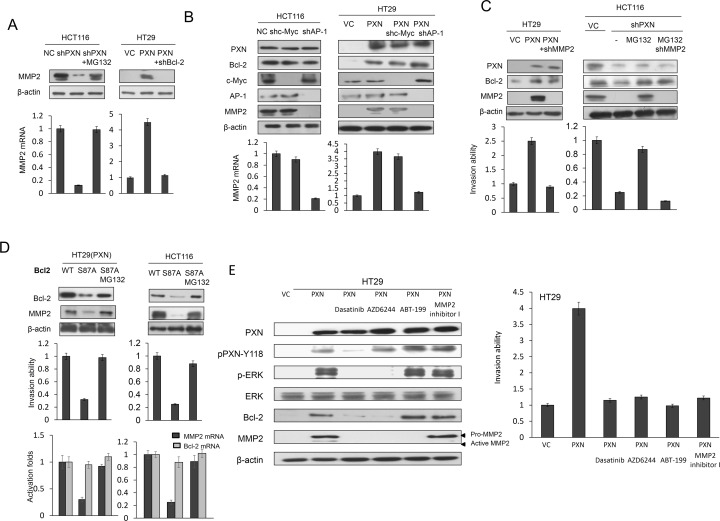
An increase in MMP2 expression induced by Bcl-2 is responsible for PXN-mediated cell invasiveness (A) HCT116 cells were transfected with PXN shRNA and then treated with 0 or 5 μmol/L MG132 for an additional 5 hours. HT29 cells were co-transfected with WT PXN and shBcl-2. Cell lysates were evaluated for MMP2 mRNA and protein expression by realtime PCR and western blotting. (B) HCT116 and PXN-overexpressing HT29 cells were transfected with c-Myc or AP-1 shRNA and cell lysates were evaluated for MMP2 mRNA and protein expression by realtime PCR and western blotting. (C) HT29 cells were transfected with indicated combinations of PXN overexpression plasmid and MMP2 shRNA. HCT116 cells were transfected with PXN and MMP2 shRNA and treated with 0 or 5 μmol/L MG132 for an additional 5 hours. These cells were evaluated for their invasion ability by Matrigel invasion assays. (D) PXN-overexpressing HT29 and HCT116 cells were transfected with WT Bcl-2 or mutant Bcl-2-S87A and then treated with 0 or 5 μmol/L MG132 for an additional 5 hours. These invasion ability of these cells was evaluated by matrigel invasion assays. MMP2 and Bcl-2 mRNA expression was evaluated by realtime PCR. (E) PXN-overexpressing HT29 cells were treated with or without Dasatinib, AZD6244, MMP2 inhibitor I, or ABT-199 for 5 hours. Cell lysates were then evaluated for MMP2 protein expression by western blotting. The indicated cells were evaluated for their invasion ability by matrigel invasion assays.

As mentioned above, upregulation of MMP2 expression is responsible for Bcl-2-mediated cell invasiveness [8, 14]. MMP2 transcriptional levels were regulated by Bcl-2 overexpression via increasing binding activity of AP-1 or c-Myc [8, 14]. We examined whether MMP2 could be responsible for PXN-mediated cell invasion by enhancement of Bcl-2 expression and protein stability. Western blotting indicated that MMP2 mRNA and protein levels were almost completely reduced in PXN-knockdown HCT116 cells and elevated in PXN-overexpressing HT29 cells (Figure 2A). Moreover, MMP2 mRNA and protein levels were almost completely reduced by AP1 shRNA transfection when compared with c-Myc shRNA transfection in HCT116 and PXN-overexpressing HT29 cells (Figure 2B). The modulation of invasion capability by PXN manipulation was consistent with the expression of Bcl-2 and MMP2 when both cell types were additionally treated with shMMP2 and/or MG132 (Figure 2C). Interestingly, MMP2, Bcl-2 expression and invasion capability decreased markedly in PXN-overexpressing HT29 cells transfected with mutant S87A Bcl-2 as compared with PXN-overexpressing HT29 cells transfected with wild-type Bcl-2. However, the decrease in MMP2 and invasion capability by S87A Bcl-2 transfection was restored by MG132 treatment (Figure 2D, upper panel). Additionally, the Bcl-2 mRNA was not changed in these transfected cells (Figure 2D, lower panel). These results clearly indicate that an increase in MMP2 expression induced by Bcl-2 protein stabilization is responsible for PXN-mediated cell invasiveness.

### Lung metastatic tumor nodules induced by a PXN-overexpressing HT29 stable clone in nude mice are nearly suppressed by Dasatinib, AZD6244, MMP2 inhibitor I, and ABT-199

**Figure 3:**
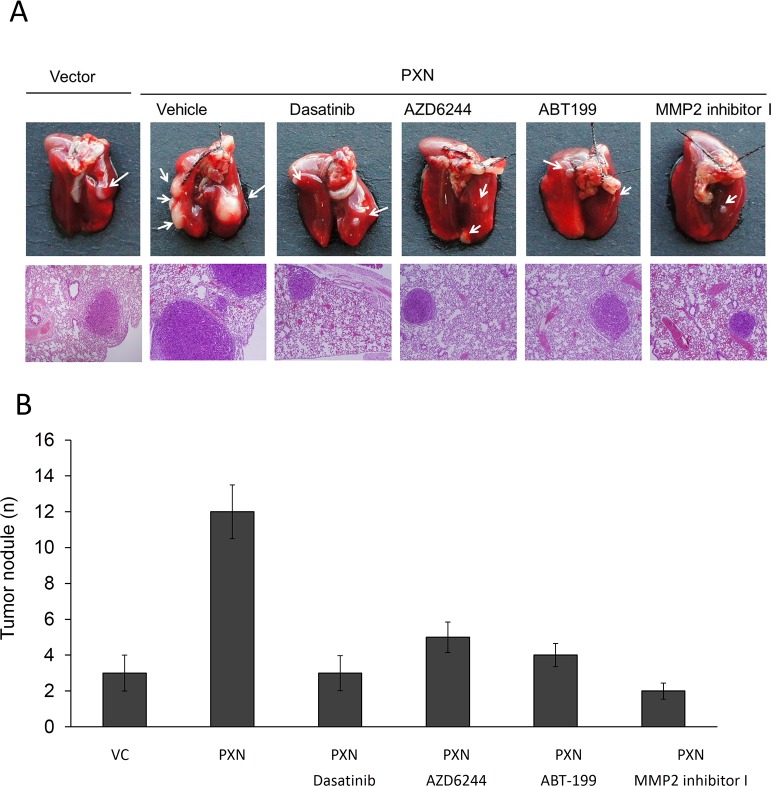
The number of lung metastatic tumor nodules in nude mice injected with PXN-overexpressing HT29 cells is strongly reduced by treatment with a Src inhibitor (Dasatinib), ERK inhibitor (AZD6244), MMP2 inhibitor I, or a Bcl-2 antagonist (ABT-199) (A) Example of lungs of mice showing visible metastases at 6 weeks after tail vein injection with the indicated cells. Representative hematoxylin and eosin staining of metastatic lung tumors from each group of mice. (B) Number of metastatic lung tumor nodules in each group of mice. Data are presented as means ± S.E.Ms.

We next examined whether Src, ERK, Bcl-2, and MMP2 inhibitors or antagonists could inhibit lung tumor nodule formation in nude mice injected with a PXN-overexpressing HT29 stable clone. We first tested the possibility that cell invasiveness mediated by PXN overexpression in HT29 cells could be modulated by Src, ERK or MMP2 inhibitor, and Bcl-2 antagonist. As shown in Figure 2E, Western blotting indicated that pPXN-Y118 expression in a PXN-overexpressing HT29 stable clone was almost completely suppressed by the Src inhibitor Dasatinib, but was unchanged by the ERK inhibitor AZD6244 (Figure 2E). In addition, the expression of pERK, Bcl-2, and MMP2 was almost completely eliminated by both Dasatinib and AZD6244, while the expression of pERK and Bcl-2 was unchanged by treatment with ABT-199 (Figure 2E). Active MMP2 expression was almost completely abolished by MMP2 inhibitor I in the stable clone. The invasion capability of a PXN-overexpressing HT29 stable clone was nearly completely reversed by Dasatinib, AZD6244, ABT-199, and MMP2 inhibitor I when compared with VC cells (Figure 2E), indicating that MMP2 expression induced by PXN overexpression is responsible for cell invasiveness in the HT29 stable clone. In animal model, the lung metastatic tumor nodules in each group of nude mice are shown in Figure 3A. The number of lung metastatic tumor nodules in each group was strongly reduced by treatment with Dasatinib, AZD6244, ABT-199, and MMP2 inhibitor I (Figure 3B). These results clearly indicate that lung tumor metastasis in nude mice may be induced by upregulation of MMP2 due to PXN-mediated stabilization of Bcl-2 protein via activation of the Src/ERK signaling pathway in the PXN-overexpressing HT29 stable clone.

### PXN was associated with Bcl-2, pBcl-2-S87, and MMP2 expression in colorectal tumors

**Figure 4:**
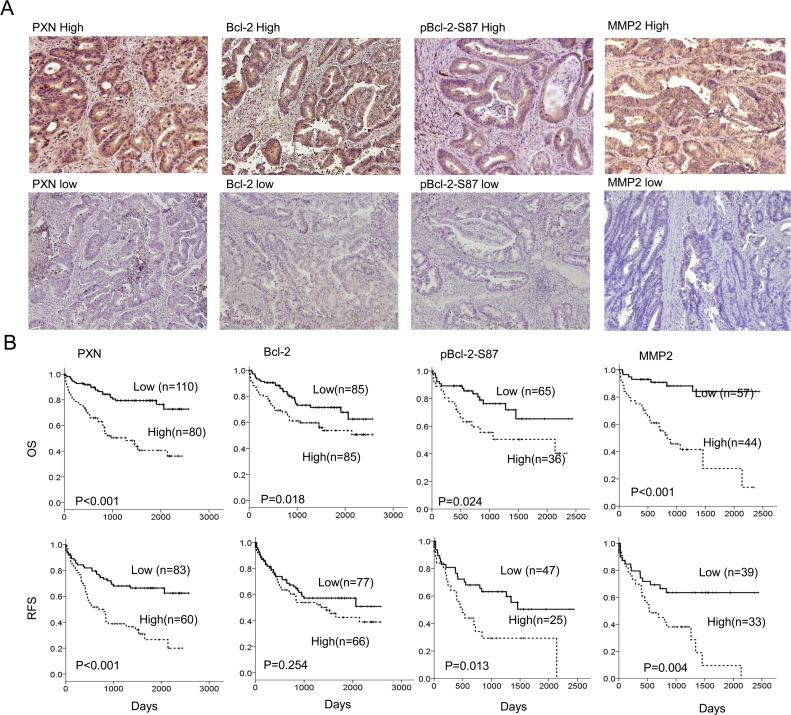
Expression of PXN, Bcl-2, pBcl-2-S87, and MMP2 in colorectal tumors is associated with reduced patient OS and RFS (A) A representative figure shows high- or low-PXN, Bcl-2, pBcl-2-S87 and MMP2 expression in colorectal cancer patients. (B) Colorectal cancer patients with tumors showing high expression of PXN, Bcl-2, pBcl-2-S87 and MMP2 had poor outcomes.

We examined the possibility that PXN expression could be associated with Bcl-2, pBcl-2-S87, and MMP2 expression in tumors from 190 colorectal cancer patients. The representative immunostaining results are shown in Figure 4A. These results indicated a greater prevalence of positive PXN, pBcl-2-S87, and MMP2 expression in advanced tumors (Duke C and D) than in early tumors (Duke A and B), but this phenomenon was not observed with Bcl-2 (P < 0.001 for PXN, P = 0.005 for pBcl-2-S87, and P < 0.001 for MMP2; Table 1). The expression of these four proteins was not associated with other clinical parameters, including age, gender, and smoking status (Table 1). Interestingly, PXN expression was positively correlated with Bcl-2, pBcl-2-S87, and MMP2 expression in colorectal tumors. A positive association was also observed between Bcl-2 and pBcl-2-S87, Bcl-2 and MMP2, and pBcl-2-S87 and MMP2 (P < 0.001 for all three pairs; Table 1). These observations from patients' tumors strongly support the mechanistic action of the cell model, indicating that PXN may promote Bcl-2 expression via increased pBcl-2-S87 by ERK activation, and in turn, upregulation of MMP2 expression.

**Table 1:** The correlation between clinical parameters with PXN, Bcl-2, pBcl-2-S87 and MMP2 expressions in colorectal cancer patients

	PXN				Bcl-2			pBcl2-S87*				MMP2*		
	No	Low	High	P	Low	High	P	No	Low	High	P	Low	High	P
**Age**														
< 66	87	54(62)	33(38)	0.284	47(54)	40(46)	0.752	46	33(72)	13(28)	0.157	28(61)	18(39)	0.411
≧ 66	103	56(54)	47(46)		58(56)	45(44)		55	32(58)	23(42)		29(53)	26(47)	
**Gender**														
Female	99	54(55)	45(45)	0.329	52(53)	47(47)	0.429	50	31(62)	18(38)	0.624	28(56)	22(44)	0.930
Male	91	56(62)	35(38)		53(58)	38(42)		51	34(67)	17(33)		29(57)	22(43)	
**Smoking status**												
Nonsmokers	138	74(54)	64(46)	0.052	75(54)	63(46)	0.679	41	48(65)	26(35)	0.860	43(58)	31(42)	0.575
Smokers	52	36(69)	16(31)		30(58)	22(42)		60	17(63)	10(37)		14(52)	13(48)	
**DUKE**												
A, B	86	62(72)	24(28)	<0.001	86(49)	37(43)	0.666	47	33(81)	8(19)	0.005	34(83)	7(17)	<0.001
C, D	104	48(46)	56(54)		56(54)	48(46)		54	32(53)	28(47)		23(38)	37(62)	
**PXN**												
Low	110				78(71)	32(29)	<0.001	110	42(89)	5(11)	<0.001	38(81)	9(19)	<0.001
High	80				27(34)	53(66)		80	23(43)	31(57)		19(35)	35(65)	
**Bcl-2**												
Low									53(100)	0(0)	<0.001	43(81)	10(19)	<0.001
High									12(25)	36(75)		19(29)	34(71)	
**pBcl-2-S87**												
Low												48(74)	17(26)	<0.001
High												9(25)	27(75)	

* 101 of 190 tumors were available for pBcl-2-S87 and MMP2 immunostaining.

### PXN, Bcl-2, pBcl-2-S87, and MMP2 expression was associated with overall survival (OS) and relapse free survival (RFS) in colorectal cancer

We next examined whether PXN, Bcl-2, pBcl-2-S87, and MMP2 expression could be associated with the outcomes of patients with colorectal cancer. Kaplan-Meier analysis indicated that patients with high-PXN, high-Bcl-2, high-pBcl-2-S87, and high-MMP2 tumors exhibited shorter OS and RFS than those with low-PXN, low-Bcl-2, low-pBcl-2-S87, and low-MMP2 tumors respectively, except that no prognostic value was evident for Bcl-2 on RFS in this population (OS: P <0.001 for PXN and MMP2, P = 0.018 for Bcl-2, P = 0.024 for pBcl-2-S87; RFS: P <0.001 for PXN, P = 0.013 for pBcl-2-S87, and P = 0.004 for MMP2; Figure 4B). The prognostic value of PXN, Bcl-2, pBcl-2-S87, and MMP2 on OS and RFS was further evidenced by Cox regression analysis, but again no prognostic value was evident for Bcl-2 on RFS (Table 2). The highest hazard ratio (HR) for OS and RFS was shown for MMP2 followed by PXN, pBcl-2-S87, and Bcl-2 (Table 2). These results suggest that an increase in pBcl-2-S87 via PXN-mediated ERK activation may promote tumor aggressiveness and poor patient outcome via increased MMP2 expression.

**Table 2:** Cox regression analysis of PXN, Bcl-2, pBcl-2-S87 and MMP2 expressions in patients with colorectal cancer.

Variables	OS				RFS			
	Case No.	Adjusted HR*	95%CI	P	Case No.	Adjusted HR*	95%CI	P
PXN								
− (Low)	110	1			83	1		
+ (High)	80	2.95	1.70–5.09	<0.001	60	2.13	1.26-3.59	0.005
Bcl-2								
− (Low)	105	1			77	1		
+ (High)	85	2	1.21–3.30	0.007	66	1.32	0.82-2.13	0.259
pBcl-2-S87								
− (Low)	65	1			47	1		
+ (High)	36	2.19	1.09–4.42	0.028	25	2.21	1.17-4.22	0.015
MMP2								
− (Low)	57	1			39	1		
+ (High)	44	5.32	2.16–13.14	<0.001	33	2.75	1.26-6.01	0.011

OS: overall survival; HR: Hazard ratio; RFS: relapse-free survival.

* HR adjusted for age, gender, smoking status, and duke stage.

## DISCUSSION

In the present study, we provided evidence from cells, xenograft tumors, and human tumors to demonstrate that ERK activation by PXN promoted tumor aggressiveness and led to poor patient outcome in colorectal cancer and that this promotion occurs via increased pBcl-2-S87-mediated MMP2 expression. These results were consistent with a previous study indicating that Bcl-2 promoted tumor invasion and lung metastasis via increased MMP2 expression [8]. To the best of our knowledge, there is for the first time to report that an increase in MMP2 expression, in response to stabilization of Bcl-2 protein stabilized by phosphorylation at Serine 87, may act as an indicator for prediction of tumors with aggressive phenotype and for poor outcome in colorectal cancer patients.

Phosphorylation of Y31 and Y118 residues of PXN is recognized as the major tyrosine phosphorylation events [15]. Phosphorylation of PXN at Y118 by FAK/Src pathway is essential for focal adhesion turnover and cell migration [16-18], and this PXN phosphorylation was frequently observed in colon cancer cells with aggressive phenotype [19]. In the present study, phosphorylation of PXN at Y31/118 is required for tumor invasion via increased Bcl-2 expression (Figure 1B and 1C). An increased in Bcl-2 expression by PXN not only prevents cell apoptosis but also promotes cell invasion (Figure 3). We thus suggest that anti-apoptosis and invasiveness concomitantly mediated by PXN overexpression may play a critical role in colorectal tumor progression and metastasis.

The role of Bcl-2 phosphorylation in tumor biology remains controversial [1-4]. MAPK/ERK in endothelial cells triggers phosphorylation of Bcl-2 at Serine 87, Threonine 56, and Threonine 74, thereby protecting it from protein degradation by ubiquitin-proteasomes, and in turn, resulting in resistance to apoptosis [7]. Bcl-2 phosphorylation at multiple sites including Serine 87 stimulates anti-apoptotic activity and also regulates entry into the cell cycle [20]. In contrast, phosphorylation of Bcl-2 at Serine 87 by the p38 MAPK pathway in embryonic fibroblasts is always associated with a decrease in the anti-apoptotic potential of Bcl-2 protein [21]. Loss of the Bcl-2 anti-apoptotic function by its phosphorylation at Serine 70 negatively correlates with tumor malignancy due to blocking of the Bcl-2 interaction with Bax, thereby resulting in favorable survival in colorectal cancer [5]. In addition, phosphorylation of Bcl-2 at Serine 87 via JNK signaling pathway promotes apoptosis in fibroblast and cancer cells when both cells are at the mitotic arrest [22, 23]. Therefore, Bcl-2 phosphorylation may implicate either increasing or decreasing its anti-apoptotic potential through different signaling pathways [24, 25]. In the present study, we provided the evidence that the stabilization of Bcl-2 by its phosphorylation at Serine 87 via PXN-mediated ERK activation promoted tumor invasion and poor patient outcome in colorectal cancer. We thus suggest that Bcl-2 phosphorylation by PXN-mediated ERK activation modulates the anti-apoptotic function and cell cycle entry and also promotes cell invasiveness via upregulation of MMP2.

As shown in Supplementary Figure 1, the cell invasion capability in HT29 cells was unchanged by transfection with WT-Bcl-2 or mutant Bcl-2-S87A; however, the invasion capability in HT29 cells transfected with mutant Bcl-2-S87A was markedly increased by a dose of MG132 treatment (0~5 μM). In addition, the cell invasion capability was also markedly elevated by transfection with a phosphorylated mutant Bcl-2-S87E mimic in HT29 cells without MG132 treatment. The invasion capability was correlated with concomitant elevation of Bcl-2 and MMP2 expression by Bcl-2-S87A+ MG132 or Bcl-2-S87E in HT29 cells. Consistent with these findings, Bcl-2 and MMP2 expression were concomitantly elevated by transfection with WT Bcl-2 or mutant Bcl-2 S87A in PXN-overexpressing HT29 cells treated with MG132. The invasion capability of PXN-overexpressing HT29 cells subjected to different treatments was dependent on the MMP2 expression levels (Figure 3D). Similar responses were also observed in HCT116 cells subjected to the same treatments (Figure 3D). Therefore, we suggest that an increased MMP2 expression by pBcl-2-S87 due to PXN-mediated ERK activation plays a crucial role in tumor invasion in colon cancer cells.

In summary, we provide evidence from cells, xenograft tumors, and human tumors to demonstrate that an increase in Bcl-2 protein stability due to its phosphorylation at Serine 87 is responsible for tumor progression and metastasis via increased MMP2 expression. We suggest that pBcl-2-S87 expression may potentially act as a biomarker for the prediction of poor survival and a high risk of tumor recurrence in patients with colorectal cancer.

## MATERIALS AND METHODS

### Study subjects

This study consisted of 190 patients with colorectal cancer. All patients were unrelated ethnic Chinese and residents of Central Taiwan. The inclusion criteria for patients were: primary diagnosed with colorectal carcinoma; no metastatic disease at diagnosis; no previous diagnosis of carcinoma; no neoadjuvant treatment before primary surgery; no evidence of disease within 1 month of primary surgery. Colorectal tumor specimens were collected by surgical resection, and surgically resected tissues were stored at 80°C at the Division of Colon and Rectum, Chung Shan Medical University Hospital (Taichung, Taiwan, ROC), between 1994 and 2006. Patients were asked to submit written informed consent and the study was approved by the Institutional Review Board (CS07159). The tumor type and stage of each collected specimen were histologically determined according to the WHO classification system. Cancer relapse data were obtained by chart review and confirmed by surgeons. Clinical parameters and overall survival (OS) data were collected from chart review and the Taiwan Cancer Registry, Department of Health, Executive Yuan, Taiwan, ROC. Survival time was defined as the period from the date of primary surgery to the date of death. The median follow-up time was 986 days (ranging from 21 to 2572 days) and the end of the follow-up period was December 2007. Based on follow-up data, relapse data from 143 patients were available, indicating that 29 patients relapsed (24 had distant metastasis, and 5 had local and distant metastasis). Tumors frequently relapsed in the liver (15 patients), metastasized in the lung (4 patients), hypopharynx (1 case) bone (1 case), left paraaortic lymph node (1 case), pelvic (1 case), rectum (1 case), and 5 patients had tumors that metastasized to more than one organ.

### Cell lines

All cells were obtained from the American Type Culture Collection (ATCC) and cultured as described. Cells were cultured and stored according to the suppliers' instructions and used at passages 5 to 20. Once resuscitated, cell lines are routinely authenticated (once every 6 months, cells were last tested in December 2011) through cell morphology monitoring, growth curve analysis, species verification by isoenzymology and karyotyping, identity verification using short tandem repeat profiling analysis, and contamination checks.

### Plasmid constructs and reagents

The PXN-GFP overexpression plasmid constructed into pAcGFP1-N1 vector was kindly provided by Dr. Salgia (The University of Chicago, USA)[26]. Mutant PXN-GFP (Tyrosine 31/118 changed to Phenylalanine: Y31/118F) and Bcl-2-Flag (Serine 87 changed to Alanine: S87A) expression constructs containing multiple-point mutations were constructed by the QuickChange site-directed mutagenesis system (Stratagene, La Jolla, CA, USA). The Bcl-2-Flag overexpression plasmid constructed into pCMV-Tag2B vector was purchased from Addgene (Addgene Company, Cambridge, MA, USA). shBcl-2 (TRCN0000010303), shc-Myc (TRCN0000039642), shAP-1 (JUN: TRCN0000338221), shMMP2 (TRCN0000051523)and shPXN (#1 TRCN0000123137; #2 TRCN0000123138) was purchased from National RNAi Core Facility, Academia Sinica, Taiwan, ROC. Different concentrations of expression plasmids were transiently transfected into colorectal cancer cells (1 × 10^6^) using the Turbofect reagent (Formentas, Glen Burnie, MD, USA). After 48 hours, the cells were harvested and whole cell extracts were assayed in subsequent experiments.

### Chemicals and antibodies

Dasatinib was obtained from LC Laboratories (Woburn, MA, USA). MMP2 inhibitor I was acquired from Santa Cruz Biotechnology (Dallas, TX, USA). AZD6244 was acquired from Selleckchem (Houston, TX, USA). ABT-199 was obtained from ActiveBiochem (Maplewood, NJ, USA). Anti-total ERK, and anti–phospho-ERK (p-ERK) antibodies were purchased from Cell Signaling (Danvers, MA, USA). Anti-PXN antibody was obtained from NeoMarker (Fremont, CA, USA). Anti-phosphoY31-PXN (pPXN-Y31), anti-c-Myc, anti-MMP2 and anti-Bcl-2 was obtained from Genetex (Irvine, CA, USA). All other antibodies were purchased from Santa Cruz Biotechnology (Dallas, TX, USA).

### Real-time quantitative RT-PCR analysis

Total RNA extraction and reverse transcriptase reaction were described previously [10]. The following primer sequences were used for amplification of the MMP2 gene: the forward primer, 5′-CGGAAAAGATTGATGCGGTATAC-3′ and the reverse primer, 5′-GTATGTCTTCTTGTTTTTGCTCCAGTTA-3′. Bcl-2 gene: the forward primer, 5′-CTGTGGATGACTGAGTACC-3′ and the reverse primer, 5′-CAGCCAGGAGAAATCAAAC-3′.

### Western blotting

The cells were lysed with lysis buffer containing 0.5% NP-40, 50 mM Tris-Cl (pH 7.5), 1 mM ethylenediaminetetraacetic acid (EDTA), and protease inhibitor cocktail (Roche, Indianapolis, IN, USA). After 3 minutes of lysis, the cell debris was removed by centrifugation, and the protein concentration was determined using a Bradford protein assay kit (Bio-Rad, Hercules, CA, USA). Equal amounts of protein were separated onto sodium dodecyl sulfate-polyacrylamide gel electrophoresis (SDS-PAGE) gels and then transferred from the gel onto a polyvinylidene difluoride membrane (PerkinElmer, Norwalk, CT, USA). After blocking, the membranes were reacted with antibody at 4°C overnight, followed by incubation with horseradish peroxidase-conjugated secondary antibody for 1 hour. The blots were observed using an enhanced chemiluminescence kit (PerkinElmer).

### Immunohistochemical analysis

IHC was used to detect PXN, pBcl-2-S87, Bcl-2 and MMP2 expression. Specimens were formalin fixed and paraffin embedded. Briefly, 3 μm sections were cut, mounted on glass, and dried overnight at 37°C. All sections were then deparaffinized in xylene, rehydrated through alcohol, and washed in phosphate-buffered saline. This buffer was used for all subsequent washes. Sections for PXN, pBcl-2-S87, Bcl-2 and MMP2 detection were heated in a microwave oven twice for 5 min in citrate buffer (pH 6.0). Anti-PXN, pBcl-2-S87, Bcl-2 and MMP2 antibody was used as the primary antibody and the incubation time was 60 min at room temperature followed by a conventional streptavidin peroxidase method (LSAB Kit K675, DAKO, Carpinteria, CA, USA). Signals were developed with 3, 3′-diaminobenzidine for 5 min and counter-stained with hematoxylin. Negative controls were obtained by leaving out the primary antibody. Metastatic lung tumor sites (lymph node) and Lymphatic cells were used as positive controls for paxillin and Bcl-2. The criteria for the determination of Bcl-2 and paxillin expression were according to a previous study [27]. The intensities of signals were evaluated independently by three observers. The levels of intensity 0–3 were established relative to the negative control (0) and strongly positive internal controls (3). Immunostaining scores were defined as the cell staining intensity (0 = nil; 1 = weak; 2 =moderate; and 3 = strong) multiplied by the percentage of labeled cells (0–100%), leading to scores from 0 to 300. A score over 150 was rated as “high” immunostaining, while a score less than 150 was rated as “low”.

### Invasion assay

A Boyden chamber with a pore size of 8 μm was used for the *in vitro* cell invasion assay. Cells (1 × 10^4^) in 0.5% serum containing culture medium (HyClone, Ogden, UT, USA) were plated in the upper chamber and 10% fetal bovine serum was added to culture medium in the lower chamber as a chemoattractant. The upper side of the filter was covered with 0.2% Matrigel (Collaborative Research, Boston, MA, USA) diluted in RPMI-1640. After 16 h, cells on the upper side of the filter were removed and cells that adhered to the underside of membrane were fixed in 95% ethanol and stained with 10% Giemsa dye. The number of invasive cells was counted. Ten contiguous fields of each sample were examined to obtain a representative number of cells that invaded across the membrane.

### *In vivo* metastasis assay

Thirty of female nude mice were randomized into group that were treated with Dasatinib (5 mg/kg/day), AZD6244 (5 mg/kg/day), ABT-199 (5 mg/kg/day), MMP2 inhibitor I (5 mg/kg/day) or its vehicle control (Saline) by intraperitoneal injection 1 week before tail vein injection. Six weeks after injection, mice were euthanized, and lungs were dissected and examined for the development of visible metastases. Mice were euthanized at 6 weeks after injection, lungs were harvested, and the number of visible surface metastases was determined. Tissues were either processed for Hematoxylin and Eosin staining.

### Statistical analysis

Statistical analysis was performed using the SPSS statistical software program (Version 18.0; SPSS Inc., Chicago, IL, USA). The association between clinical parameters and protein expressions was analyzed by the chi-square test. Multivariate Cox regression analysis was performed to determine overall survival (OS) and relapse-free survival (RFS). The analysis was stratified for all known variables (age, gender, smoking status, and tumor stage) and protein expressions.

## SUPPLEMENTARY MATERIAL AND FIGURE



## References

[1] Manne U, Weiss HL, Grizzle WE (2000). Bcl-2 expression is associated with improved prognosis in patients with distal colorectal adenocarcinomas. International journal of cancer Journal international du cancer.

[2] Bosari S, Moneghini L, Graziani D, Lee AK, Murray JJ, Coggi G, Viale G (1995). bcl-2 oncoprotein in colorectal hyperplastic polyps, adenomas, and adenocarcinomas. Human pathology.

[3] Bhatavdekar JM, Patel DD, Ghosh N, Chikhlikar PR, Trivedi TI, Suthar TP, Doctor SS, Shah NG, Balar DB (1997). Coexpression of Bcl-2, c-Myc, and p53 oncoproteins as prognostic discriminants in patients with colorectal carcinoma. Diseases of the colon and rectum.

[4] Sinicrope FA, Hart J, Michelassi F, Lee JJ (1995). Prognostic value of bcl-2 oncoprotein expression in stage II colon carcinoma. Clinical cancer research: an official journal of the American Association for Cancer Research.

[5] Kondo E, Miyake T, Shibata M, Kimura T, Iwagaki H, Nakamura S, Tanaka T, Ohara N, Ichimura K, Oka T, Yanai H, Shibasaki F, Yoshino T (2005). Expression of phosphorylated Ser70 of Bcl-2 correlates with malignancy in human colorectal neoplasms. Clinical cancer research: an official journal of the American Association for Cancer Research.

[6] Shitashige M, Toi M, Yano T, Shibata M, Matsuo Y, Shibasaki F (2001). Dissociation of Bax from a Bcl-2/Bax heterodimer triggered by phosphorylation of serine 70 of Bcl-2. Journal of biochemistry.

[7] Breitschopf K, Haendeler J, Malchow P, Zeiher AM, Dimmeler S (2000). Posttranslational modification of Bcl-2 facilitates its proteasome-dependent degradation: molecular characterization of the involved signaling pathway. Molecular and cellular biology.

[8] Choi J, Choi K, Benveniste EN, Rho SB, Hong YS, Lee JH, Kim J, Park K (2005). Bcl-2 promotes invasion and lung metastasis by inducing matrix metalloproteinase-2. Cancer research.

[9] Brown MC, Turner CE (2004). Paxillin: adapting to change. Physiological reviews.

[10] Wu DW, Wu TC, Wu JY, Cheng YW, Chen YC, Lee MC, Chen CY, Lee H (2014). Phosphorylation of paxillin confers cisplatin resistance in non-small cell lung cancer via activating ERK-mediated Bcl-2 expression. Oncogene.

[11] Yin H, Zhang Q, Wang X, Li T, Wan Y, Liu Y, Zhu J (2014). Role of paxillin in colorectal carcinoma and its relationship to clinicopathological features. Chinese medical journal.

[12] Chen DL, Wang DS, Wu WJ, Zeng ZL, Luo HY, Qiu MZ, Ren C, Zhang DS, Wang ZQ, Wang FH, Li YH, Kang TB, Xu RH (2013). Overexpression of paxillin induced by miR-137 suppression promotes tumor progression and metastasis in colorectal cancer. Carcinogenesis.

[13] Wu DW, Huang CC, Chang SW, Chen TH, Lee H (2014). Bcl-2 stabilization by Paxillin confers 5-Fluorouracil Resistance in Colorectal Cancer Cell Death and Differentiation.

[14] Lu Q, Hong W (2009). Bcl2 enhances c-Myc-mediated MMP-2 expression of vascular smooth muscle cells. Cellular signalling.

[15] Bellis SL, Miller JT, Turner CE (1995). Characterization of tyrosine phosphorylation of paxillin *in vitro* by focal adhesion kinase. The Journal of biological chemistry.

[16] Schaller MD, Parsons JT (1995). pp125FAK-dependent tyrosine phosphorylation of paxillin creates a high-affinity binding site for Crk. Molecular and cellular biology.

[17] Su S, Li Y, Luo Y, Sheng Y, Su Y, Padia RN, Pan ZK, Dong Z, Huang S (2009). Proteinase-activated receptor 2 expression in breast cancer and its role in breast cancer cell migration. Oncogene.

[18] Vindis C, Teli T, Cerretti DP, Turner CE, Huynh-Do U (2004). EphB1-mediated cell migration requires the phosphorylation of paxillin at Tyr-31/Tyr-118. The Journal of biological chemistry.

[19] Sachdev S, Bu Y, Gelman IH (2009). Paxillin-Y118 phosphorylation contributes to the control of Src-induced anchorage-independent growth by FAK and adhesion. BMC cancer.

[20] Deng X, Gao F, Flagg T, May WS (2004). Mono- and multisite phosphorylation enhances Bcl2's antiapoptotic function and inhibition of cell cycle entry functions. Proceedings of the National Academy of Sciences of the United States of America.

[21] De Chiara G, Marcocci ME, Torcia M, Lucibello M, Rosini P, Bonini P, Higashimoto Y, Damonte G, Armirotti A, Amodei S, Palamara AT, Russo T, Garaci E, Cozzolino F (2006). Bcl-2 Phosphorylation by p38 MAPK: identification of target sites and biologic consequences. The Journal of biological chemistry.

[22] Maundrell K, Antonsson B, Magnenat E, Camps M, Muda M, Chabert C, Gillieron C, Boschert U, Vial-Knecht E, Martinou JC, Arkinstall S (1997). Bcl-2 undergoes phosphorylation by c-Jun N-terminal kinase/stress-activated protein kinases in the presence of the constitutively active GTP-binding protein Rac1. The Journal of biological chemistry.

[23] Wang TH, Wang HS, Ichijo H, Giannakakou P, Foster JS, Fojo T, Wimalasena J (1998). Microtubule-interfering agents activate c-Jun N-terminal kinase/stress-activated protein kinase through both Ras and apoptosis signal-regulating kinase pathways. The Journal of biological chemistry.

[24] Blagosklonny MV (2001). Unwinding the loop of Bcl-2 phosphorylation. Leukemia.

[25] Blagosklonny MV (2001). Paradox of Bcl-2 (and p53): why may apoptosis-regulating proteins be irrelevant to cell death?. BioEssays: news and reviews in molecular, cellular and developmental biology.

[26] Jagadeeswaran R, Surawska H, Krishnaswamy S, Janamanchi V, Mackinnon AC, Seiwert TY, Loganathan S, Kanteti R, Reichman T, Nallasura V, Schwartz S, Faoro L, Wang YC, Girard L, Tretiakova MS, Ahmed S (2008). Paxillin is a target for somatic mutations in lung cancer: implications for cell growth and invasion. Cancer research.

[27] Viard-Leveugle I, Veyrenc S, French LE, Brambilla C, Brambilla E (2003). Frequent loss of Fas expression and function in human lung tumours with overexpression of FasL in small cell lung carcinoma. The Journal of pathology.

