# Supplementary material for: Paxillin promotes colorectal tumor invasion and poor patient outcomes via ERK-mediated stabilization of Bcl-2 protein by phosphorylation at Serine 87
Source: Oncotarget. 2015 Mar 12;6(11):8698–708. doi: 10.18632/oncotarget.3537 (PMC4496177; doi:10.18632/oncotarget.3537)
Supplement: Supplementary file 1 [file oncotarget-06-8698-s001.pdf]

Paxillin promotes colorectal tumor invasion and poor patient outcomes via ERK-mediated stabilization of Bcl-2 protein by phosphorylation at Serine 87

Supplementary Material

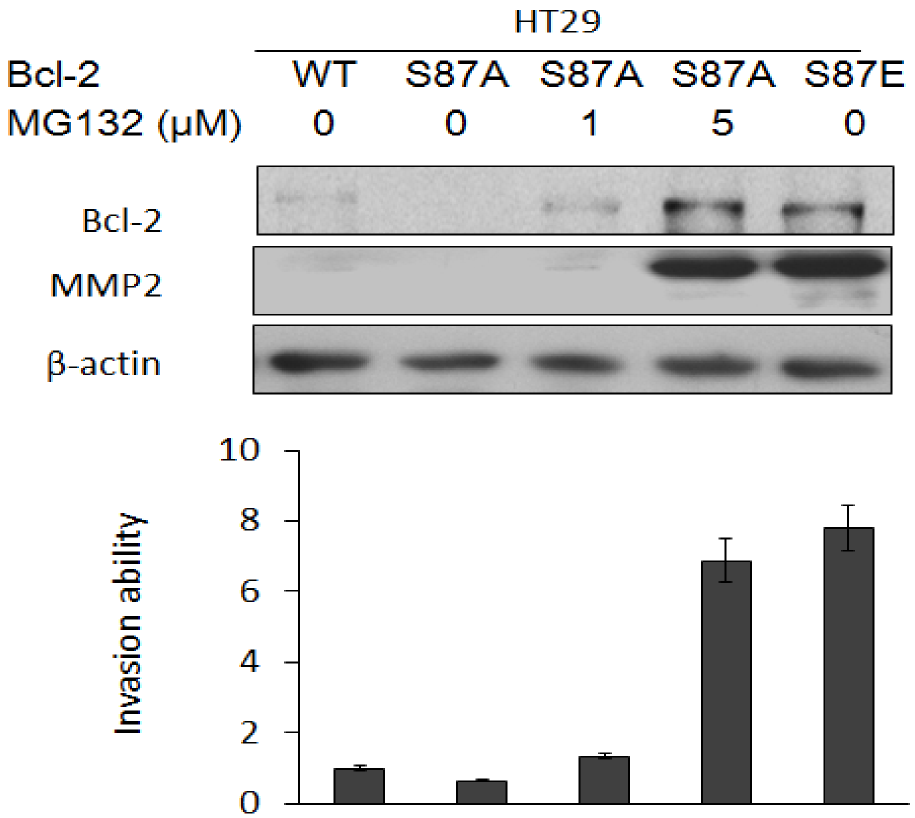

Supplementary Figure 1: HT29 cells were transfected with WT-Bcl-2 or mutant Bcl-2-S87A and Bcl-2-S87E and then treated with 0~5 μmol/L MG132 for an additional 5 hours. The cells were analyzed cell invasion capability by matrigel invasion assay.
